# How Do Intergenerational Economic Support, Emotional Support and Multimorbidity Affect the Catastrophic Health Expenditures of Middle-Aged and Elderly Families?–Evidence From CHARLS2018

**DOI:** 10.3389/fpubh.2022.872974

**Published:** 2022-04-08

**Authors:** Shaoliang Tang, Ling Yao, Zhengjun Li, Tongling Yang, Meixian Liu, Ying Gong, Yun Xu, Chaoyu Ye

**Affiliations:** ^1^School of Health Economics and Management, Nanjing University of Chinese Medicine, Nanjing, China; ^2^Institute of Traditional Chinese Medicine, Nanjing University of Chinese Medicine, Nanjing, China

**Keywords:** intergenerational economic support, emotional support, multimorbidity, catastrophic health expenditure, middle-aged, elderly family

## Abstract

**Objectives:**

The elderly face multiple vulnerabilities such as health, economy and society, and are prone to catastrophic health expenditures. This study aims to analyze the impact of children's intergenerational economic support, emotional support, and illness on the catastrophic health expenditures of middle-aged and elderly families.

**Methods:**

Using China Health and Retirement Longitudinal Study (CHARLS 2018) data to calculate the catastrophic health expenditure of Chinese households as the dependent variable. Taking children's intergenerational economic support, emotional support and multimorbidity as core independent variables, gender, age, marital status, medical insurance and other variables as control variables, and perform logistic regression analysis. According to the heterogeneity analysis of age and gender, the impact of intergenerational economic support, emotional support and multimorbidity on the catastrophic health expenditure of middle-aged and elderly families is explored.

**Results:**

When catastrophic health expenditures occur in middle-aged and elderly families, the children's intergenerational economic support will increase significantly, especially in families with members aged 60–74. Children's emotional support can effectively reduce the risk of catastrophic health expenditures for middle-aged and elderly families. Compared with children's intergenerational economic support and emotional support, the impact of multimorbidity on the catastrophic health expenditures of middle-aged and elderly families is the most significant. Suffering from multimorbidity can increase the risk of catastrophic health expenditures for middle-aged and elderly families, especially families with male members suffering from multiple diseases.

**Conclusions:**

It is recommended that we should do a good job in popularizing the knowledge of chronic diseases to minimize the occurrence of multimorbidity. The government should establish group medical insurance related to chronic disease diagnosis. According to the severity of the disease or the special circumstances of the patient, the level of medical insurance reimbursement is divided in detail, especially for chronic disease clinics and drug reimbursement. Children should be encouraged to strengthen the emotional connection and effective care of the elderly, focusing on the elderly 60–74 years old, in order to reduce their care pressure and maintain the physical and mental health of the elderly.

## Introduction

### Chronic Diseases and Catastrophic Health Expenditure (CHE)

At present, chronic diseases have become one of the major public health problems affecting human health. According to statistics from the World Health Organization (WHO), chronic diseases such as heart disease, stroke, cancer, chronic respiratory diseases and diabetes are by far the world's leading cause of death, accounting for 63% of all deaths. With the rapid development of China's economy, industrialization, accelerated urbanization, and changes in the disease spectrum, chronic diseases have become a major health problem for Chinese residents. Incurable chronic diseases such as high blood pressure, diabetes, stroke and coronary heart disease not only lead to a decline in human health, but also cause catastrophic health expenditures for the family, leading to poverty and return to poverty due to illness (1, 2). Among them, the same individual suffering from two or more chronic diseases at the same time can be called multimorbidity.

Scholars have launched a lot of research on chronic diseases and catastrophic health expenditures. Somkotra et al. (3) found that families with a high proportion of elderly members, families with chronic diseases or disabilities among members, and families with hospitalized members are more likely to have catastrophic medical expenditures. Wang et al. (4) found that the economic burden of chronic diseases in rural Malawi is very high, causing severe and catastrophic expenditures and exacerbating poverty. In addition, Jiang et al. (5) evaluated the catastrophic health expenditures of families with chronic diseases in different types of insurance in China. The results showed that 10.53% of families with chronic diseases fell into poverty due to medical expenditures, which was more than twice the proportion of families without chronic diseases. Xu et al. (6) studied the impact of China's new medical reform on their catastrophic health expenditures by taking the families of patients with chronic diseases as the research object. They found that the implementation of the new medical reform did not effectively reduce their catastrophic medical expenditures. In addition, Dugee et al. (7) found that compared with other families, families with members suffering from chronic diseases and multimorbidities are more likely to experience catastrophic expenditure and medical poverty. Although Mongolia's population has a high health insurance coverage rate, health expenditures still have a significant impact on poverty, especially for families with chronic disease patients. Fu et al. (8) compared the differences in catastrophic health expenditures (CHE) between urban and rural families with chronic diseases in China. They found that compared with urban households with chronic diseases, the risk of catastrophic health expenditures (CHE)in rural Chinese households with chronic diseases is higher, and the economic burden of disease is heavier. Brinda et al. (9), Si et al. (10), and Hailemichael et al. (11) specifically studied the family disasters of patients with chronic non-communicable diseases such as diabetes, respiratory diseases, gastrointestinal diseases, dementia, hypertension and depression. It is found that these families bear a higher risk of catastrophic health expenditure. Zhou et al. (12) found that members suffering from chronic non-communicable diseases are one of the important factors for catastrophic health expenditures in the family. Kien et al. (13) assessed the socioeconomic inequality of catastrophic health expenditures and poverty related to non-communicable diseases in northern Vietnam. They found that family self-reported diagnosis of noncommunicable diseases had the greatest correlation with catastrophic health expenditures and poverty.

We found that scholars have proved through a large number of empirical studies that chronic diseases can significantly increase the possibility of catastrophic health expenditures for families. Secondly, scholars take the families of patients with chronic diseases as specific research objects and directly study the catastrophic health expenditures of the families of patients with chronic diseases. Some scholars have carried out comparative studies to compare the catastrophic health expenditures of different regions and families of patients with chronic diseases and those with non-chronic diseases. Some scholars study chronic diseases as an influencing factor of catastrophic health expenditures. Some scholars have carried out research on the types of chronic diseases. In summary, the current research on chronic diseases and single chronic diseases has been in-depth. However, due to work pressure, living habits and other factors, modern people, especially the elderly, are increasingly susceptible to many chronic diseases (14). Therefore, it is of great significance to further study the catastrophic health expenditure of the multimorbidity, especially the elderly for multimorbidity.

### Intergenerational Support and Catastrophic Health Expenditure (CHE)

Intergenerational relationship refers to the relationship between two adjacent generations, the core of which is the parent-child relationship (15). Children's intergenerational support refers to the help provided by adult children to older parents, such as emotional support, financial assistance, and informal care. Scholars have also conducted a series of studies on intergenerational support. Merz et al. (16) studied whether the quality of intergenerational relations regulates the happiness between parents and adult children. They found that the well-being of parents benefited from the support of their children. Chen et al. (17) investigated the differences between children's intergenerational support and life satisfaction of the elderly in different Chinese elderly groups. The results show that there is an age difference between children's intergenerational support and life satisfaction of the elderly, whether it is exchange mode or different types of support. Schwarz et al. (18) compared samples from rural and urban areas in China, Indonesia and Germany. It is found that under different cultural backgrounds, the happiness of elderly mothers is differently related to the help they give to their adult daughters. Yang et al. (19) used 78 Asian female college students as samples to explore the relationship between intergenerational cultural conflicts, parental social support and subjective well-being. In addition, Wu et al. (20) studied 153 elderly people in Taiwan Province of China and found that depressive symptoms are significantly related to social media use, social support, and intergenerational relationships. Choi et al. (21) investigated the relationship between intergenerational support patterns and depressive symptoms of elderly men and women in South Korea and found that lack of mutual financial support significantly increased the risk of depressive symptoms. Gierveld et al. (22) used the data of the intergenerational and gender surveys in three countries in Eastern Europe and two countries in Western Europe to study the relationship between intergenerational support and loneliness. Some scholars have focused on the differences in intergenerational support. For example, Chen et al. (23) studied the gender differences in intergenerational support between Chinese only-child families and multi-child families. Song et al. (24) used a random effects model to explore the gender differences in intergenerational support for the elderly in rural Chinese families. Bordone et al. (25) compared the intergenerational support between international immigrants and non-immigrant populations in northern, central and southern Europe. Moore et al. (26) explored the difference in intergenerational support between divorced adults and their parents. Santarelli et al. (27) analyzed the similarities and differences between parent-child relationship, closeness and support in four selected Italian regions (Liguria, Umbria, Sicily, and Sardinia). Zhou et al. (28) examined the impact of taking care of grandchildren on the health of grandparents and the role of intergenerational support for adult children.

Combining the existing literature, we found that scholars mainly studied the relationship between intergenerational support and satisfaction, happiness or intergenerational support and loneliness and depression. Some scholars have carried out research on the differences in intergenerational support between different regions and roles. Only a few scholars pay attention to the relationship between intergenerational support and health, and the research conclusions have not yet reached a consensus (29). Intergenerational support is an important link between parents and adult children. In some countries, most elderly people rely on their adult children for financial and instrumental support. At the same time, older parents are also an important source of family help and child's care for adult children. This enables adult children to better participate in the labor market. These intergenerational relationships are the cornerstone of the family's economic welfare and also protect each generation from age-related health threats (30). Therefore, the introduction of intergenerational support into the study of health or catastrophic health expenditure is of great significance to further clarify the relationship between the two. This paper innovatively explores the impact of intergenerational economic support, intergenerational emotional support and multimorbidity on the catastrophic health expenditures of middle-aged and elderly families, which not only provides new ideas for research in this field, but also provides new methods for alleviating catastrophic health expenditures.

## Methods

### Data Source

The data used in this article comes from the 2018 China Health and Retirement Longitudinal Study (CHARLS). CHARLS is a large-scale national follow-up survey designed and implemented by the Social Science Investigation Center of Peking University specifically for middle-aged and elderly people. The main target of the CHARLS survey is the elderly in China. The survey aims to establish a high-quality micro-database representing the families and individuals of middle-aged and elderly people aged 45 and above in China. The content of the survey includes information from the macro-social and economic conditions to the micro-level personal health conditions. CHARLS adopted a multi-stage sampling method with probability proportional to scale. The survey samples covered middle-aged and elderly populations in 28 provinces, municipalities, and autonomous regions across the country. Since 2011, CHARLS has officially launched a national survey. The survey followed the baseline survey sample in 2013. The CHARLS follow-up survey covered 450 villages in 150 counties and districts across the country. A total of 23,000 respondents from approximately 12,400 households successfully accepted the interview. On the whole, the sample can represent the middle-aged and elderly population in China and has excellent representation. This article first matched the household codes and obtained a total of 9,110 household samples to calculate catastrophic health expenditures. After excluding the missing values of key variables and control variables, 4,184 effective samples were obtained.

### Dependent Variable

The explanatory variable in this article is whether the household has catastrophic health expenditures. Due to the strong rigidity of food expenditures, in order to avoid deviations in the measurement of catastrophic health expenditures of low-income families, this paper uses non-food expenditures as the denominator to measure catastrophic health service expenditures, and household health expenditures as the numerator to examine family health Expenditure accounts for the remaining proportion of household effective income after deducting basic consumption expenditure (31). Use *E*_*i*_ to indicate whether catastrophic health expenditures occur, and the calculation formula is as follows:


(1) $$\begin{array}{l} E_{i}=\;\{\begin{array}{c} 0\;if\;T_{i}/(x_{i}-f(x))<z\\ 1\;if\;T_{i}/(x_{i}-f(x))\geq z \end{array}\; \end{array}$$

In the formula, *T*_*i*_ is the family's annual health expenditure. *x*_*i*_ is the annual household consumption expenditure. *f*(*x*) is the household food consumption expenditure. *z* is the threshold. With reference to the WHO definition criteria, this article assumes that *z* = 40% (32). The incidence of catastrophic health expenditure refers to the percentage of households with catastrophic health expenditures in all households. According to *E*_*i*_, the incidence of family catastrophic health expenditure can be calculated. This article uses the three questions CE010_6, GE000_W4 and GE006_W4 in the CHARLS2018 questionnaire to calculate the incidence of catastrophic health expenditures. CE010_6 is the direct or indirect medical expenditures of your family in the past year. Indirect medical expenditures refer to transportation expenses, nutrition expenses, family care expenses, etc. incurred due to medical treatment. It does not include the part already paid by medical insurance. GE000_W4 is how much money your family spends a month on average (rent, food, clothing, communications, utilities, fuel, service expenses, entertainment expenses, daily necessities and medical expenses are included). GE006_W4 is how much your family spent on food in the last week (wine display, banquets, dining out, buying cigarettes, drinks, etc. are not included). Use “0” to indicate that the family has not incurred catastrophic health expenditures, and “1” indicates that the family has incurred catastrophic health expenditures.

### Independent Variables

The core independent variables of this article are intergenerational economic support, intergenerational emotional support and multimorbidity. The intergenerational economic support and emotional support in this article refer to the support provided by adult children to their parents. Many scholars have discussed the main content of intergenerational support provided by offspring (33–35). Existing literature and theories generally summarize the intergenerational support provided by children to the elderly into three aspects: economic support, emotional support and life care (36). This article only specifically studies two aspects of intergenerational economic support and emotional support. The data of intergenerational economic support comes from CE002_1 in the CHARLS 2018 questionnaire. CE002_1 is how much economic support you or your spouse have received from your children in the past year. This paper uniformly adds 1 to the economic support in the empirical process and then takes the logarithm to avoid the influence of extreme values, missing values and zero values. The emotional support data comes from DD006_W4 in the CHARLS2018 questionnaire. DD006_W4 is how often you and your children saw each other in the past year. Assign a value of “1” to see almost once a day. Assign a value of “2” to see almost once a week. Assign a value of “3” to see almost once a month. Assign a value of “4” to meet infrequently and a value of “5” to almost never meet.

The chronic disease data in the CHARLS2018 questionnaire includes hypertension, dyslipidemia, diabetes (abnormal blood sugar), malignant tumors, chronic lung disease, liver disease, heart disease, kidney disease, stomach or digestive system disease, arthritis, asthma, etc. 14 Kind of chronic disease. The multimorbidity referred to in this article refers to the group suffering from 2 or more chronic diseases in the questionnaire. “0” means not suffering from multimorbidity. “1” means suffering from multimorbidity.

### Covariates

Considering the influence of other factors on intergenerational economic support, intergenerational emotional support and multimorbidity, this paper incorporates variables such as age, gender, marital status, education level, medical insurance, ADL, intergenerational care, and self-rated health (SRH) into the regression model as covariates to reduce endogeneity and heteroscedasticity (29). Among them, age is a numeric variable. The data comes from the respondents' answers to the question “What is your true date of birth?” in the CHARLS2018 questionnaire. The gender is derived from the interviewer's record. “1” means male and “2” means female. Marital status comes from the question “What is your current marital status?”. “1” means married and living with your spouse, “2” means married, but has not lived with your spouse temporarily due to work and other reasons, “3” means separation (no longer living together as a spouse), “4” means Divorced, “5” means widowed and “6” means never married. The level of education comes from the question “Your highest degree?”. The items are in the order of 7 levels from illiterate to doctoral degree. Since the samples are mainly middle-aged and elderly, and the generally accepted education level is not high, this article sets “illiterate, not graduated from elementary school” as “beginner” with a value of 1, and “graduated from elementary school” as “intermediate” with a value of 2. And finally, set “junior high school and above” to “advanced” and assign a value of 3 (37). The medical insurance data comes from the question “Are you currently participating in the following medical insurance?”.

There are 12 options from urban employee medical insurance to no insurance. Due to the small participation in public medical care and medical aid, this paper assigns urban employee medical insurance to 1, urban and rural resident medical insurance to 2, urban resident medical insurance to 3, new rural cooperative medical insurance to 4, and public medical insurance to medical aid, etc., are assigned a value of 5 as other insurances. The most widely used measures of self-care ability are the Katz Index Scale of Activities of Daily Life (ADL) and the Lawton Instrumental Activities of Daily Life (IADL) (38, 39). This article uses the ADL scale to measure the self-care ability of the elderly as one of the covariates. ADL includes the ability to dress, bathe, eat, get in and out of bed, get up/squat while going to the toilet, and control the bowel movement (40). If any of them is difficult, assign a value of “1”. If there is no difficulty in all 6 items, the value is “0”. The ADL data comes from question DB010 to question DB015. Intergenerational care refers to the elderly taking care of their grandchildren. The data comes from the question “Did you or your spouse spend time looking after your grandchildren and grandchildren in the past year?”, “1” means yes, and “2” means no. Self-rated health (SRH) comes from the question “What do you think of your health?”. There are five options from “very good” to “very bad”, with values of 1–5 in turn. The detailed description of all variables is shown in Table 1.

**Table 1:** Description of variables.

**Variable name**	**Definition**	**Obs**
CHE	0 = No 1 = Yes	4,184
Economic support	Numerical variable	4,184
Emotional support	1 = Almost every day 2 = Almost every week 3 = Almost every month 4 = Seldom 5 = Hardly	4,184
Multimorbidity	0 = No 1 = Yes	4,184
Age	Numerical variable	4,184
Gender	1 = Male 2 = Female	4,184
Marital status	1 = Married with spouse present 2 = Married but not living with spouse 3 = Separated 4 = Divorced 5 = Widowed 6 = Never married	4,184
Education	1 = Primary school or below 2 = Primary-school graduated 3 = Middle school and above	4,184
Insurance	1 = Urban employee medical insurance 2 = Urban and rural resident medical insurance 3 = Urban resident medical insurance 4 = New rural cooperative medical insurance 5 = other	4,184
ADL	0 = No difficulty 1 = Have difficulty with any of them	4,184
Grandchild Care (GC)	0 = No 1 = Yes	4,184
SRH	1 = Very good 2 = Good 3 = Fair 4 = Poor 5 = Very poor	3,918

### Methodology

In this study, the dependent variable catastrophic health expenditure and the independent variable multimorbidity are binary variables, and the independent variable intergenerational economic support is an ordered multi-categorical variable. Therefore, this paper chooses Logit model for empirical analysis.


(2) $$\begin{array}{l} Logit(Y_{i})=Logit(\frac{P}{1-P})=\alpha_{0}+\alpha_{1}IES_{i}+\alpha_{2}IAS_{i}\;\\ \;+\alpha_{3}Mul_{i}+\alpha_{4}X_{i}+\varepsilon \end{array}$$

Among them, *Y*_*i*_ represents the possibility of catastrophic health expenditure in household I; *IES*_*i*_ is intergenerational economic support for children; *Mul*_*i*_ is whether any member of the family i suffers from multiple diseases; *X*_*i*_ is the control variable; ε is a random disturbance item.

## Results

### Descriptive Statistics Results

Table 2 shows the basic characteristics of the surveyed objects in this article, and they are divided by region. Physically, women account for the majority of the surveyed population, accounting for 60% of the total population. Nearly half of the population is 60–74 years old. People aged 75 and above account for about 20% of the total population. The married population accounts for more than half of the total population. Due to the low level of education in China before the 1980s, more than 50% of the elderly had an education level lower than that of elementary school. It is worth noting that the proportion of people with a junior high school education level and above in the western region is significantly lower than that in the eastern and central regions, indicating that there is a certain degree of educational inequity in China. From the perspective of residence, most of the respondents are located in rural areas, which is more than 70% of the total population. Although most of the investigators have medical insurance, most of them are new rural cooperative medical insurance. Nearly 30% of middle-aged and elderly people have difficulties with ADL. Nearly 80% of people with SRH are average and below. Nearly 30% of middle-aged and elderly people have taken on the task of inter-generational care. More than 30% of the population is suffering from catastrophic health expenditures, and the western region has the highest proportion of catastrophic health expenditures. More than 20% of the population suffers from a variety of chronic diseases, and the central region has the highest prevalence of disease in the population. Less than 30% of middle-aged and elderly people have emotional connection with their children almost every day. The level of economic support for the elderly is also relatively low, with an overall average value of only 4820.36 RMB. Per capita economic support is the highest in the eastern region, at 5649.46 RMB. There is a big difference between the central region, western region and the eastern region, with only 4793.74 RMB and 3975.71 RMB. The differences in economic support for elderly patients with chronic diseases in the three places also reflect the regional differences in China's economic development.

**Table 2:** Descriptive statistics.

**Proportion (%)**	**All (*N* = 4,184)**	**East (*N* = 1,305)**	**Central (*N* = 1,650)**	**West (*N* = 1,229)**
**CHE**				
No	67.76	67.36	68.42	67.29
Yes	32.24	32.64	31.58	32.71
**Multimorbidity**				
No	77.75	80.46	75.33	78.11
Yes	22.25	19.54	24.67	21.89
**Emotional support (EmoSup)**				
Almost every day	24.78	28.74	24.24	21.32
Almost every week	22.23	27.82	20.61	18.47
Almost every month	23.16	22.91	22.00	24.98
Seldom	27.08	18.77	30.12	31.81
Hardly	2.75	1.76	3.03	3.42
**Gender**				
Male	38.10	37.62	38.36	38.24
Female	61.90	62.38	61.64	61.76
**Age**				
45–59	28.75	25.36	32.12	27.83
60–74	47.63	49.81	46.12	47.36
75 and above	23.61	24.83	21.76	24.82
**Marital status**				
Married	59.27	60.07	61.58	55.33
Separated/divorced/windowed/never married	40.73	39.93	38.42	44.67
**Education**				
Primary school or below	52.63	51.80	48.36	59.24
Primary-school graduated	21.85	21.30	21.70	22.62
Middle school and above	25.53	26.90	29.94	18.14
**Place of residence**				
City or town central areas	18.40	16.02	23.39	14.24
Town or semi-rural areas	6.88	5.52	9.09	5.37
Rural areas	74.71	78.47	67.52	80.39
**Medical insurance**				
Urban employee medical insurance	12.36	13.56	14.18	8.62
Urban and rural resident medical insurance	12.19	18.77	7.94	10.90
Urban resident medical insurance	4.76	2.68	7.70	3.01
New rural cooperative medical insurance	68.67	62.15	68.42	75.92
Other	2.03	2.84	1.76	1.55
**ADL**				
No difficulty	70.58	73.41	69.94	68.43
Have difficulty with any of them	29.42	26.59	30.06	31.57
**Grandchild care (GC)**				
No	72.47	75.33	72.42	69.49
Yes	27.53	24.67	27.58	30.51
**SRH**				
Very good	6.81	9.90	6.56	3.91
Good	9.21	9.32	9.46	8.77
Fair	46.78	49.34	45.95	45.23
Poor	28.36	25.00	28.96	31.08
Very poor	8.83	6.44	9.07	11.02
**ln(Economic support) (RMB/year)**				
Mean	4820.36	5649.46	4793.74	3975.71

**Figure 1:**
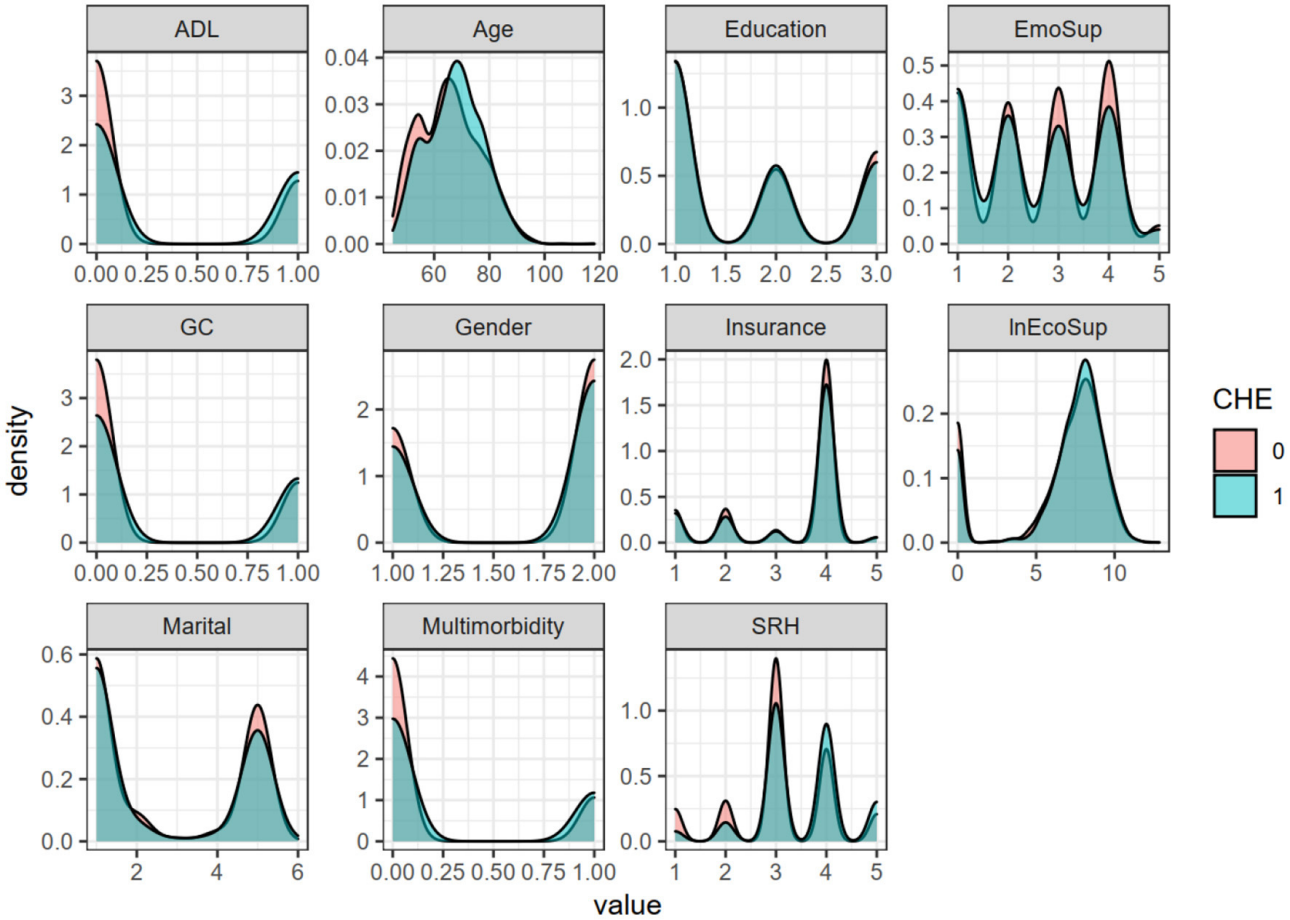
Density curves of respective variables under different catastrophic health expenditures.

It can be seen from Figure 1 that among variables such as multimorbidity, intergenerational emotional support, and ADL, the occurrence of family catastrophic health expenditures is more obvious. In variables such as education level and medical insurance, the occurrence of catastrophic health expenditures for households is not very obvious.

### Stepwise Regression Results

**Figure 2:**
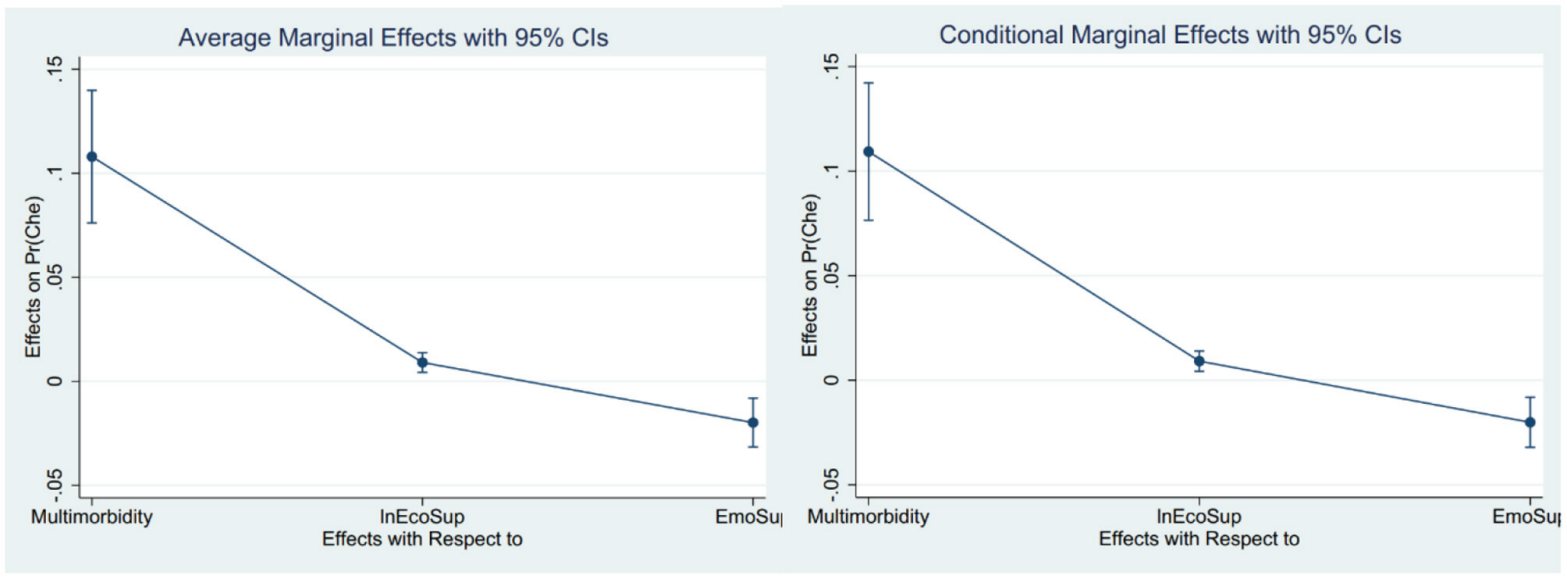
Visualization of the results of the marginal effect analysis of the core independent variables.

**Figure 3:**
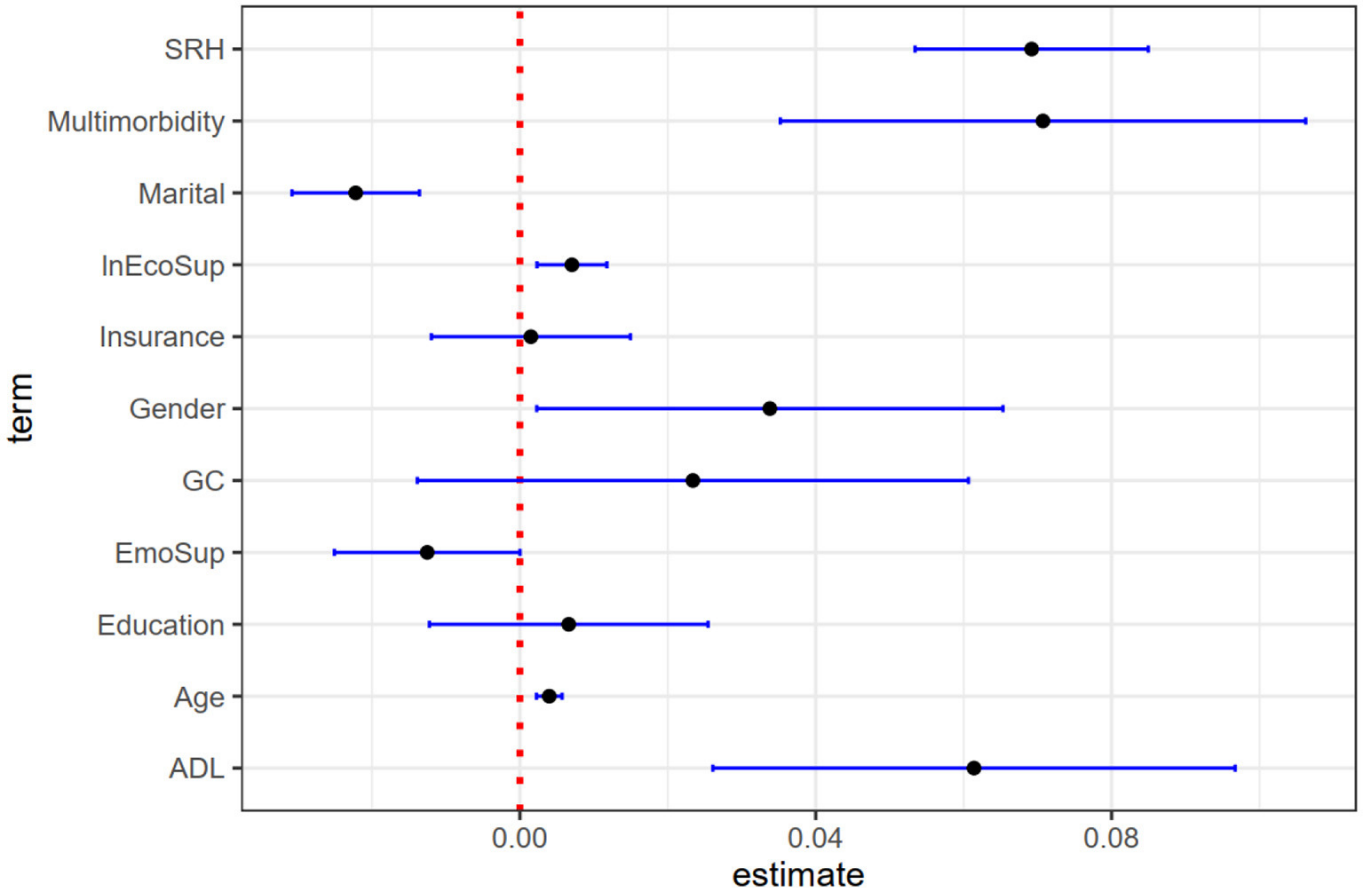
Visualization of logistic regression coefficients.

There are two main parts to the stepwise regression. In the first part, bring the core independent variables and dependent variables into the model (Table 3, Model 1–3) and perform marginal effect analysis (Figure 2, Table 2), excluding the control variables. In the second part, the control variables are gradually included, and the changes in the relationship between the core independent variables and the dependent variables are observed again (Table 3, Model 4–8). Table 3 shows all the results of the stepwise regression. Table 4 and Figure 2 show the results of the marginal effect analysis of the core independent variables. In addition, Figure 3 visualizes the regression coefficients. It can be seen initially from Figure 3 that the three core independent variables, intergenerational economic support, intergenerational emotional support, and multimorbidity, all have a significant impact on catastrophic health expenditures, and multimorbidity has the most significant impact. The confidence interval for the coefficients of the control variables insurance, education level, and intergenerational care includes 0, so the impact of these variables on catastrophic health expenditures is not statistically significant.

**Table 3:** Stepwise regression results (*z* = 0.4).

**Vars**	**Models**							
	**1**	**2**	**3**	**4**	**5**	**6**	**7**	**8**
lnEcoSup	0.0470^***^ (0.0112)	0.0428^***^ (0.0113)	0.0425^***^ (0.0113)	0.0342^***^ (0.0115)	0.0335^***^ (0.0115)	0.0340^***^ (0.0116)	0.0342^***^ (0.0116)	0.0370^***^ (0.0121)
EmoSup		−0.0951^***^ (0.0278)	−0.0960^***^ (0.0280)	−0.0610^**^ (0.0293)	−0.0633^**^ (0.0294)	−0.0671^**^ (0.0296)	−0.0667^**^ (0.0296)	−0.0646^**^ (0.0310)
Multimorbidity			0.496^***^ (0.0769)	0.504^***^ (0.0774)	0.509^***^ (0.0775)	0.455^***^ (0.0784)	0.444^***^ (0.0786)	0.314^***^ (0.0837)
Gender				0.146^**^ (0.0745)	0.149^**^ (0.0745)	0.126^*^ (0.0750)	0.118 (0.0751)	0.163^**^ (0.0785)
Age				0.0194^***^ (0.0037)	0.0200^***^ (0.00373)	0.0160^***^ (0.00379)	0.0147^***^ (0.00385)	0.0195^***^ (0.00423)
Marital status				−0.101^***^ (0.0204)	−0.102^***^ (0.0205)	−0.107^***^ (0.0206)	−0.107^***^ (0.0206)	−0.106^***^ (0.0214)
Education				−0.0172 (0.0428)	−0.00174 (0.0447)	0.0149 (0.0449)	0.0223 (0.0452)	0.0321 (0.0468)
Insurance					0.0373 (0.0315)	0.0258 (0.0317)	0.0227 (0.0318)	0.00601 (0.0332)
ADL						0.473^***^ (0.0742)	0.424^***^ (0.0790)	0.276^***^ (0.0837)
GC							0.153^*^ (0.0833)	0.0864 (0.0864)
SRH								0.347^***^ (0.0407)
Obs	4,184	4,184	4,184	4,184	4,184	4,184	4,184	3,918

*Standard errors in parentheses*,

*** *p < 0.01*,

** *p < 0.05*,

* *p < 0.1*.

**Table 4:** The results of the analysis of the marginal effects of the core independent variables.

**CHE**	**Margin**	**Delta-method Std. Err**.	**z**	**P > |z|**	**[95% Conf. interval]**	
Multimorbidity	0.1079809	0.0162625	6.64	0.000	0.076107	0.1398548
lnEcoSup	0.0090274	0.0024238	3.72	0.000	0.0042768	0.0137779
EmoSup	−0.0198584	0.0060003	−3.31	0.001	−0.0316189	−0.008098
**CHE**	**Margin at means**	**Delta-method Std. Err**.	**z**	**P** **>** **|z|**	**[95% Conf. interval]**	
Multimorbidity	0.109326	0.0167444	6.53	0.000	0.0765075	0.1421445
lnEcoSup	0.0091398	0.002464	3.71	0.000	0.0043104	0.0139692
EmoSup	−0.0201058	0.0139692	−3.30	0.001	−0.0320559	−0.0081558

#### The Influence of Intergenerational Economic Support on Catastrophic Health Expenditure

From the models 1–3 in Table 3, we can see that intergenerational economic support has a significant positive impact on the incidence of family catastrophic health expenditures (COA: 0.043, COA = coefficient of action; *P* < 0.01). That is, when intergenerational economic support increases, the incidence of catastrophic health expenditures in the family will increase significantly. After including the control variables, the results remained stable. Through the analysis of marginal effects, it can be seen that under the condition that other influencing factors remain unchanged, increasing intergenerational economic support will increase the incidence of family catastrophic health expenditure by 0.90%; When other influencing factors are averaged, increasing intergenerational economic support will increase the incidence of family catastrophic health expenditure by 0.91% (Table 4, Figure 3).

#### The Impact of Intergenerational Emotional Support on Catastrophic Health Expenditures

From the models 1–3 in Table 3, it shows that intergenerational emotional support has a significant negative impact on the incidence of family catastrophic health expenditure (COA: −0.096, COA = coefficient of effect; *P* < 0.01). That is, the more emotionally connected children are, the less likely the family will experience catastrophic health expenditures. After including the control variables, the results remained stable. Through the marginal effect analysis, it can be seen that under the condition that other influencing factors remain unchanged, improving emotional support will reduce the incidence of family catastrophic health expenditure by 1.99%;When other influencing factors are averaged, improving emotional support can reduce the incidence of family catastrophic health expenditure by 2.10% (Table 4, Figure 3).

#### The Impact of Multimorbidity on Catastrophic Health Expenditures

From the models 1–3 in Table 3, we can see that suffering from multimorbidity has a significant positive impact on the incidence of family catastrophic health expenditures (COA: −0.496, COA = coefficient of action; *P* < 0.01). It shows that families with multimorbidity are more prone to catastrophic health expenditures. After including the control variables, the results remained stable. From the marginal effect analysis, it can be seen that under the condition that other influencing factors remain unchanged, suffering from multimorbidity will increase the incidence of catastrophic health expenditures by 10.64%. When other influencing factors are averaged, suffering from multimorbidity will increase the incidence of catastrophic health expenditures by 10.77%.

#### The Impact of Covariates on Catastrophic Health Expenditures

From the models 4–8 in Table 3, we can see that gender, age, ADL and self-rated health (SRH) have a significant positive impact on family catastrophic health expenditures (COA: 0.163, 0.0195, 0.276, 0,347, COA = coefficient of action; *P* < 0.05, *P* < 0.01, *P* < 0.01, *P* < 0.01). It shows that with age, the incidence of family catastrophic health expenditures gradually increases. Compared with men, women have a higher incidence of catastrophic health expenditures. Respondents who have difficulties in any of the ADL activities in their families have a higher incidence of catastrophic health expenditures. The higher the SRH level (the worse the health status), the higher the incidence of catastrophic health expenditures in the family. Education level, medical insurance and inter-generational care have a positive impact on family catastrophic health expenditures (COA: 0.032, 0.006, 0.086), but the results did not pass the significance test. Marital status has a significant negative impact on family catastrophic health expenditures (COA: −0.106, COA = coefficient of action; *P* < 0.01), indicating that unmarried health expenditures are more likely to lead to catastrophic health expenditures than married ones.

### Robustness Test

In the previous logistic regression, this article calculated the catastrophic health expenditure with *z* = 0.4. In order to verify the robustness of the results, this paper once again calculates catastrophic health expenditures with *z* = 0.2, *z* = 0.3, and *z* = 0.5, and re-analyses the regression analysis. The specific results are in Tables 1–3 in the Appendix section. The results show that the core independent variables intergenerational economic support and multimorbidity still have a significant positive impact on catastrophic health expenditures, and intergenerational emotional support has a significant negative impact on catastrophic health expenditures. The influence of control variables such as gender and age on catastrophic health expenditure is basically consistent with the original regression results. It can be seen that the regression results of this article are robust.

### Heterogeneity Validation

Table 5 reports the difference analysis results of age and gender. Model 1–6 divides the age into three groups according to the middle-aged and old age classification standards (Group 1: 45 ≤ *age* ≤ 59; Group 2: 60 ≤ *age* ≤ 74; Group 3: *age* ≥ 75). Then regresses them separately. Models 1, 3, and 5 are the core independent variable regressions, and models 2, 4, and 6 are the results after incorporating the control variables. The results show that intergenerational economic support has a positive impact on family catastrophic health expenditures, and it is more significant in group 2 (60 ≤ *age* ≤ 74) (COA: 0.0627, COA = coefficient of action, *P* < 0.01). The impact of intergenerational affective support on catastrophic health expenditures in group 1 (45 ≤ *age* ≤ 59) did not pass the significance test, but it has a significant negative impact on catastrophic health expenditures in group 2 (60 ≤ *age* ≤ 74) and group 3 (*age* ≥ 75) (COA: 0.119,0.146, COA=coefficient of action; *P* < 0.01, *P* < 0.05). Multimorbidity has a positive impact on the catastrophic health expenditures of the three groups, but only the group 2 has a statistically significant result (COA: 0.598, COA = coefficient of action; *P* < 0.01).

**Table 5:** Heterogeneity analysis results.

**Vars**	**Models**									
	**Age** **=** **1**		**Age** **=** **2**		**Age** **=** **3**		**Gender** **=** **1**		**Gender** **=** **2**	
	**1**	**2**	**3**	**4**	**5**	**6**	**7**	**8**	**9**	**10**
lnEcoSup	0.0299 (0.0184)	0.0285 (0.0189)	0.0662^***^ (0.0180)	0.0627^***^ (0.0187)	−0.00959 (0.0262)	0.0102 (0.0306)	0.0467^***^ (0.0179)	0.0403^**^ (0.0193)	0.0399^***^ (0.0146)	0.0333^**^ (0.0156)
EmoSup	0.0660 (0.0583)	0.0355 (0.0605)	−0.110^***^ (0.0411)	−0.119^***^ (0.0427)	−0.154^**^ (0.0615)	−0.146^**^ (0.0716)	-0.116^**^ (0.0456)	−0.0882^*^ (0.0505)	-0.0826^**^ (0.0355)	−0.0486 (0.0394)
Multimorbidity	0.133 (0.159)	−0.0760 (0.167)	0.775^***^ (0.108)	0.598^***^ (0.114)	0.266^*^ (0.159)	0.00990 (0.196)	0.605^***^ (0.124)	0.383^***^ (0.137)	0.429^***^ (0.0980)	0.271^**^ (0.106)
Gen		0.0638 (0.148)		0.0901 (0.109)		0.429^**^ (0.185)				
Age								0.0165^**^ (0.00678)		0.021^***^ (0.00544)
MS		−0.0856^*^ (0.0507)		−0.0464^*^ (0.0276)		−0.217^***^ (0.0453)		−0.186^***^ (0.0384)		−0.072^***^ (0.0264)
Edu		0.0460 (0.0836)		0.0323 (0.0661)		0.0141 (0.115)		−0.0291 (0.0719)		0.0702 (0.0618)
Ins		0.115 (0.0722)		−0.0123 (0.0469)		−0.0765 (0.0652)		0.0211 (0.0505)		−0.00258 (0.0444)
ADL		0.221 (0.182)		0.371^***^ (0.114)		0.167 (0.173)		0.157 (0.143)		0.337^***^ (0.104)
GC		0.264 (0.200)		0.105 (0.123)		0.0543 (0.172)		0.224 (0.154)		0.0284 (0.109)
SRH		0.331^***^ (0.0774)		0.354^***^ (0.0581)		0.323^***^ (0.0874)		0.371^***^ (0.0669)		0.330^***^ (0.0515)
Obs	1,203	1,187	1,993	1,941	988	790	1,594	1,512	2,590	2,406

*Standard errors in parentheses*,

*** *p < 0.01*,

** *p < 0.05*,

* *p < 0.1*.

Model 7–10 is divided into two groups according to gender (male group: gender = 1; female group: gender = 2), and regression is performed respectively. Model 7 and Model 9 are the core independent variable regressions, and Model 8 and Model 10 are the results after incorporating the control variables. The results show that intergenerational economic support has a positive impact on family catastrophic health expenditures, and it is more significant among male (COA:0.0403, COA = coefficient of action, *P* < 0.05). Intergenerational emotional support has a negative impact on family catastrophic health expenditures, and the impact is also more significant among male (COA:−0.0882, COA=coefficient of action, *P* < 0.05). In the female group, after including the control variables, the impact of intergenerational economic support on family catastrophic health expenditures was not statistically significant. Multimorbidity have a positive impact on catastrophic health expenditures, and the impact is more significant in male (COA: 0.383, COA = coefficient of action, *P* < 0.01).

## Discussion

Through regression analysis and marginal effect analysis, it can be seen that whether or not suffering from multimorbidity has a significant positive impact on family catastrophic health expenditures, and it is more significant than intergenerational economic support and intergenerational emotional support. This means that families with multimorbidity are more likely to fall into catastrophic health expenditures than those without multimorbidity. This result is consistent with previous studies (41, 42). Gao Meng-ting et al. also found that the risk of catastrophic health expenditure in families with chronic patients increased by 1.06 times (43). Chronic diseases have a long course and are generally difficult to cure. Many chronic diseases have resulted in higher drug and medical costs. For example, a study by Agudelo et al. (2) found that in Medellin, Colombia, out-of-pocket expenditures related to high blood pressure accounted for 1.6% of the total annual household basic expenditures on average in terms of purchasing power parity (USD-PPP). In addition, China's medical insurance policy pays less attention to chronic diseases in outpatient clinics, and the coordination mechanism for chronic diseases in outpatient clinics in various regions has not been established and perfected (1, 44). However, the ceiling line for outpatient chronic disease management in each coordinating unit is relatively low. Once a family member has multimorbidity, the outpatient chronic disease co-ordination system cannot effectively alleviate the risk of spillover economic losses, which makes these families more prone to catastrophic health expenditures.

Increases in intergenerational economic support are often accompanied by increases in the incidence of household catastrophic health expenditures. China has always attached importance to filial piety. Affected by traditional concepts such as “bring up children for the purpose of being looked after in old age”, the old-age care methods in China, especially in rural areas, are mainly family care and back-feeding. The quality of life and health of the rural elderly directly depend on the intergenerational support provided by their children. The research of Jia et al. and Zhang et al. (45, 46) also proved this view. However, in order to reduce the burden on children, the elderly generally rarely ask for financial support from their children when they have sufficient pension funds or in a timely manner. When catastrophic health expenditures occur in middle-aged and elderly families, children have to increase economic support to ease the pressure on parents. This is manifested as a positive relationship between economic support and catastrophic health expenditure (46). On the contrary, older people are eager to have more emotional connections with their children because of their loneliness. Therefore, the more frequent and close the emotional connection between children and their parents, the better the health of the parents (45). This is often accompanied by a reduction in household medical spending. The risk of households falling into catastrophic health expenditure is also reduced.

Heterogeneity analysis has found that the increase in intergenerational economic support is most likely to lead to catastrophic health expenditures for families with 60–74-year-olds. The study by Zhang et al. (1) also proves that the probability and average intensity of catastrophic health expenditure risk in families with elderly people over 65 are significantly higher. Compared with middle-aged people aged 45–59, the elderly is at higher risk of illness due to reduced (degenerate) physical function, resistance and immunity, and are more likely to have catastrophic health expenditures. However, compared with the elderly over the age of 75, the elderly aged 60–74 still have better activities and working ability, and can provide assistance in housework and child care for adult children. Therefore, should they suffer from catastrophic health expenditures, their children must also provide greater financial support. In addition, families with male members with multiple chronic diseases are more likely to fall into catastrophic health expenditures. The study of Zhai et al. (47) also found that men are more likely to fall into poverty due to illness than women. China's ideology of “Men take charge of the outside, and women take charge of the inside” is deeply rooted, so generally speaking, males are the main labor force in China, especially in rural families (48). When the main labor force in the family is affected by the multimorbidity, and the economic income is greatly reduced or even disappeared, it is very easy to fall into catastrophic health expenditure.

And unexpectedly, enrolling in health insurance can exacerbate household catastrophic health expenditures. This is consistent with the research of Zhao et al. and Huang et al. (44, 49). We believe that the “currency illusion” created by medical insurance makes insured people seek higher-priced medical and health services, leading to increased out-of-pocket medical expenses, which in turn increases the economic burden of disease. On the other hand, there are differences in the socioeconomic characteristics of different types of medical insurance groups. The insured population of Urban resident medical insurance and Urban employee medical insurance is generally the urban population, who receive stable income and a high level of medical reimbursement. However, the new rural cooperative medical insurance participants are rural people with lower income levels and reimbursement levels, which makes them more vulnerable to the economic burden of disease (50).

## Limitation

This study also has some limitations. First, the intergenerational support studied in this paper only includes two dimensions of economic support and emotional support, and does not include life care, so it is not comprehensive enough. Second, this study only focused on the incidence of catastrophic health expenditures and did not measure their intensity, and the findings may not be rich enough. It is hoped that it can be further improved in the future research.

## Conclusion

The findings suggest that intergenerational economic support increases significantly when middle-aged and older households experience catastrophic health expenditure, especially for households with members aged 60–74. Emotional support can mitigate the risk of catastrophic health expenditure. Compared with the former two, multimorbidity has a more prominent exacerbating effect on household catastrophic health expenditure, especially for families with multimorbidty male members. In addition, participation in health insurance may also increase the risk of household catastrophic health expenditures due to the “currency illusion” of health insurance and differences in the socioeconomic characteristics of the insured population. Therefore, it is suggested that we should do a good job in the popularization of chronic disease knowledge to minimize the occurrence of multimorbidity. Secondly, we should set up group medical insurance related to the diagnosis of chronic diseases and divide the medical insurance reimbursement level in detail according to the severity of the disease or the special situation of the patient, especially in terms of chronic disease outpatient clinics and drug reimbursement. In addition, the medical insurance department need to promote the reform of medical insurance and ensure the rational distribution of medical insurance funds among different types of insurance. Also, adult children are encouraged to strengthen their emotional connection and effective care of the elderly. Another important point is that the elderly aged 60–74 should be focused on. The children ought to reduce the pressure of their intergenerational care and maintain the physical and mental health of the elderly.

## Data Availability Statement

Publicly available datasets were analyzed in this study. This data can be found here: http://charls.pku.edu.cn/.

## Author Contributions

ST provided guidance on the thinking the study. LY conceived and wrote the full text. ZL provided us with the full text of the embellishment. TY cleaned data on economic support and intergeneration care. ML painted Figure 3. YG, YX, and CY provided suggestions for revisions to the article. All authors read and approved the final manuscript.

## Funding

Support for this study was provided by National Natural Science Foundation of China (71673148), National Natural Science Foundation of China (72074125), and Priority Academic Program Development of Jiangsu Higher Education Institutions (2019YSHL072).

## Conflict of Interest

The authors declare that the research was conducted in the absence of any commercial or financial relationships that could be construed as a potential conflict of interest.

## Publisher's Note

All claims expressed in this article are solely those of the authors and do not necessarily represent those of their affiliated organizations, or those of the publisher, the editors and the reviewers. Any product that may be evaluated in this article, or claim that may be made by its manufacturer, is not guaranteed or endorsed by the publisher.

## Abbreviations

CHE: Catastrophic Health Expenditure
EcoSup: Economic Support
lnEcoSup: ln(Economic Support)
EmoSup: Emotional Support
ADL: Katz Index Scale of Activities of Daily Life
GC: Grandchild Care
SRH: Self-Rated Health.
