# Supplementary material for: How Do Intergenerational Economic Support, Emotional Support and Multimorbidity Affect the Catastrophic Health Expenditures of Middle-Aged and Elderly Families?–Evidence From CHARLS2018
Source: Front Public Health. 2022 Apr 8;10:872974. doi: 10.3389/fpubh.2022.872974 (PMC9024169; doi:10.3389/fpubh.2022.872974)
Supplement: Supplementary file 1 [file Data_Sheet_1.pdf]

Appendix

Table 1 Robustness test results($z = 0.2$)

Vars	Models							
	1	2	3	4	5	6	7	8
**lnEcoSup**	0.0554***	0.0509***	0.0508***	0.0433***	0.0432***	0.0436***	0.0438***	0.0455***
	(0.0103)	(0.0104)	(0.0105)	(0.0107)	(0.0107)	(0.0107)	(0.0107)	(0.0112)
**EmoSup**		-0.104***	-0.105***	-0.0731***	-0.0734***	-0.0766***	-0.0763***	-0.0769***
		(0.0262)	(0.0264)	(0.0276)	(0.0277)	(0.0278)	(0.0278)	(0.0292)
**Multimorbidity**			0.520***	0.529***	0.529***	0.483***	0.472***	0.349***
			(0.0749)	(0.0754)	(0.0755)	(0.0762)	(0.0765)	(0.0815)
**Gender**				0.187***	0.187***	0.167**	0.161**	0.193***
				(0.0702)	(0.0702)	(0.0706)	(0.0707)	(0.0738)
**Age**				0.0190***	0.0191***	0.0157***	0.0146***	0.0185***
				(0.00349)	(0.0035)	(0.0036)	(0.0036)	（0.0040）
**Marital status**				-0.114***	-0.114***	-0.119***	-0.119***	-0.122***
				(0.0193)	(0.0193)	(0.0194)	(0.0194)	(0.0202)
**Education**				0.0100	0.0119	0.0267	0.0332	0.0394
				(0.0402)	(0.0420)	(0.0423)	(0.0424)	(0.0439)
**Insurance**					0.00455	-0.00572	-0.00841	-0.0321
					(0.0297)	(0.0299)	(0.0299)	(0.0312)
**ADL**						0.426***	0.383***	0.253***
						(0.0717)	(0.0761)	(0.0809)
**GC**							0.136*	0.0749
							(0.0799)	(0.0857)
**SRH**								0.330***
								(0.0379)
**Obs**	4184	4184	4184	4184	4184	4184	4184	3918

Standard errors in parentheses， *** p<0.01, ** p<0.05, * p<0.1

Table 2 Robustness test results($z = 0.3$)

Vars	Models							
	1	2	3	4	5	6	7	8
**lnEcoSup**	0.0503***	0.0456***	0.0453***	0.0364***	0.0363***	0.0367***	0.0369***	0.0392***
	(0.0108)	(0.0109)	(0.0109)	(0.0111)	(0.0111)	(0.0112)	(0.0112)	(0.0117)
**EmoSup**		-0.109***	-0.110***	-0.0722**	-0.0724**	-0.0759***	-0.0756***	-0.0737**
		(0.0270)	(0.0272)	(0.0285)	(0.0285)	(0.0287)	(0.0287)	(0.0301)
**Multimorbidity**			0.503***	0.509***	0.510***	0.461***	0.450***	0.326***
			(0.0755)	(0.0761)	(0.0762)	(0.0769)	(0.0772)	(0.0822)
**Gender**				0.130*	0.130*	0.109	0.102	0.136*
				(0.0722)	(0.0723)	(0.0727)	(0.0728)	(0.0759)
**Age**				0.0216***	0.0216***	0.0181***	0.0168***	0.0221***
				(0.0036)	(0.0036)	(0.0037)	(0.0037)	(0.0041)
**Marital status**				-0.108***	-0.108***	-0.113***	-0.113***	-0.113***
				(0.0199)	(0.0199)	(0.0200)	(0.0200)	(0.0208)
**Education**				0.0130	0.0144	0.0297	0.0368	0.0483
				(0.0414)	(0.0433)	(0.0436)	(0.0438)	(0.0453)
**Insurance**					0.00350	-0.00729	-0.0103	-0.0255
					(0.0305)	(0.0306)	(0.0307)	(0.0320)
**ADL**						0.437***	0.389***	0.233***
						(0.0728)	(0.0774)	(0.0821)
**GC**							0.148*	0.0911
							(0.0814)	(0.0869)
**SRH**								0.333***
								(0.0393)
**Obs**	4184	4184	4184	4184	4184	4184	4184	3918

Standard errors in parentheses, *** p<0.01, ** p<0.05, * p<0.1

Table 3 Robustness test results($z = 0.5$)

Vars	Models							
	1	2	3	4	5	6	7	8
**lnEcoSup**	0.0437***	0.0401***	0.0397***	0.0319***	0.0308**	0.0314***	0.0316***	0.0341***
	(0.0117)	(0.0117)	(0.0118)	(0.0120)	(0.0120)	(0.0121)	(0.0121)	(0.0127)
**EmoSup**		-0.0823***	-0.0830***	-0.0504*	-0.0544*	-0.0588*	-0.0582*	-0.0574*
		(0.0289)	(0.0291)	(0.0304)	(0.0305)	(0.0307)	(0.0307)	(0.0323)
**Multimorbidity**			0.490***	0.499***	0.508***	0.445***	0.430***	0.300***
			(0.0790)	(0.0795)	(0.0796)	(0.0806)	(0.0810)	(0.0861)
**Gender**				0.155**	0.160**	0.133*	0.123	0.166**
				(0.0774)	(0.0774)	(0.0780)	(0.0782)	(0.0817)
**Age**				0.0178***	0.0188***	0.0142***	0.0125***	0.0170***
				(0.0038)	(0.0039)	(0.0039)	(0.0040)	(0.0044)
**Marital**				-0.0964***	-0.0979***	-0.104***	-0.104***	-0.101***
				(0.0212)	(0.0212)	(0.0214)	(0.0214)	(0.0222)
**Education**				-0.0356	-0.00907	0.0107	0.0200	0.0356
				(0.0444)	(0.0464)	(0.0467)	(0.0470)	(0.0487)
**Insurance**					0.0649**	0.0517	0.0477	0.0369
					(0.0330)	(0.0332)	(0.0333)	(0.0348)
**ADL**						0.553***	0.490***	0.325***
						(0.0763)	(0.0813)	(0.0860)
**GC**							0.194**	0.124
							(0.0857)	(0.0911)
**SRH**								0.368***
								(0.0426)
**Obs**	4184	4184	4184	4184	4184	4184	4184	3918

Standard errors in parentheses, *** p<0.01, ** p<0.05, * p<0.1
